# Resolvin D1 Reverts Lipopolysaccharide-Induced TJ Proteins Disruption and the Increase of Cellular Permeability by Regulating I****κ****B****α**** Signaling in Human Vascular Endothelial Cells

**DOI:** 10.1155/2013/185715

**Published:** 2013-12-05

**Authors:** Xingcai Zhang, Tingting Wang, Ping Gui, Chengye Yao, Wei Sun, Linlin Wang, Huiqing Wang, Wanli Xie, Shanglong Yao, Yun Lin, Qingping Wu

**Affiliations:** ^1^Department of Anesthesiology, Union Hospital, Tongji Medical College, Huazhong University of Science and Technology, Wuhan 430022, China; ^2^Department of Anesthesiology, Ningbo First Hospital, Ningbo 315010, China

## Abstract

Tight Junctions (TJ) are important components of paracellular pathways, and their destruction enhances vascular permeability. Resolvin D1 (RvD1) is a novel lipid mediator that has treatment effects on inflammatory diseases, but its effect on inflammation induced increase in vascular permeability is unclear. To understand whether RvD1 counteracts the lipopolysaccharide (LPS) induced increase in vascular cell permeability, we investigated the effects of RvD1 on endothelial barrier permeability and tight junction reorganization and expression in the presence or absence of LPS stimulation in cultured Human Vascular Endothelial Cells (HUVECs). Our results showed that RvD1 decreased LPS-induced increased in cellular permeability and inhibited the LPS-induced redistribution of zo-1, occludin, and F-actin in HUVECs. Moreover, RvD1 attenuated the expression of I**κ**B**α** in LPS-induced HUVECs. The NF-**κ**B inhibitor PDTC enhanced the protective effects of RvD1 on restoration of occludin rather than zo-1 expression in LPS-stimulated HUVECs. By contrast, the ERK1/2 inhibitor PD98059 had no effect on LPS-induced alterations in zo-1 and occludin protein expressions in HUVECs. Our data indicate that RvD1 protects against impairment of endothelial barrier function induced by LPS through upregulating the expression of TJ proteins in HUVECs, which involves the I**κ**B**α** pathway but not the ERK1/2 signaling.

## 1. Introduction

Endothelial cells form a selective barrier that dynamically controls the transport of bioactive molecules between the circulating blood and the interstitial fluid [1, 2]. The disruption of this barrier induces a direct increase in vascular permeability. Vascular permeability is determined by a combination of transcellular and paracellular pathways, with the latter being a major contributor to inflammation-induced barrier disruption [3]. Studies have shown that lipopolysaccharide (LPS), by eliciting a variety of inflammatory response, can induce the breakdown of endothelial barrier functions. However, the underlying mechanism is unclear, and the potential interventions are required to reverse the inflammation-induced barrier disruption.

Tight junctions (TJ) are important components of paracellular pathways, and their destruction causes barrier hyperpermeability. TJ proteins are located at the apical-most portion of the lateral interendothelial membrane. Occludin is a major transmembrane protein localizing at the TJ [4]. Zonula occludens 1 (zo-1) is considered as a scaffolding protein, linking TJ transmembrane proteins to cytoskeletal filaments. Studies have shown that zo-1 is required for occludin to be localized at TJ. Disrupting either the expression or the distribution of zo-1 leads to disruption of TJ assembly [5–7]. It has also been shown that zo-1 limits solute transport, by depleting zo-1 in MDCK cells [5]. These investigations suggest that TJ proteins occludin and zo-1 play active roles in regulating paracellular permeability of endothelia [8].

Resolvin D1 (RvD1) is a novel lipid mediator that has been identified to possess the property in resolving inflammatory exudates. It is enzymatically derived from docosahexaenoic acid (DHA) [9, 10]. RvD1 has important beneficial effects in the treatment of many inflammatory diseases. It markedly reduces the levels of IL-1*β* and IL-6 and increases the levels of IL-10 and IFN-*γ* [11]. Pretreatment with RvD1 reduces lung edema and inhibits the activation of ERK1/2 in an acute lung injury model of mice [12]. Moreover, some studies show that activation of the MAPK extracellular signal-regulated kinase (ERK) 1/2 (p44/p42, resp.) is associated with the disruption of TJ proteins [13, 14]. Interestingly, RvD1 significantly reduces tumor necrosis factor (TNF)-*α* induced phosphorylation of I*κ*B, a critical regulator of NF-*κ*B activation and nuclear translocation in human monocytes [15].

Therefore, in the present study, we tested the hypothesis that RvD1 could counteract the LPS-induced increase in permeability, primarily through reversing LPS-induced TJ proteins disruption and expression in human umbilical vein endothelial cells (HUVECs).

## 2. Materials and Methods

### 2.1. Cell Cultures and Treatments

HUVECs were obtained from ATCC. Cells were cultured in M199 media with 10% fetal bovine serum (FBS), 100 U/mL penicillin, and 100 *μ*g/mL streptomycin. HUVECs were incubated at 37°C in a humidified atmosphere of 5% CO2. After 2-3 days, cells reached 80–90% confluence in all experiments.

HUVECs were randomly divided into four groups: (1) control group: cells without treatment; (2) LPS group: cells were treated with LPS (400 ng/mL) for 6 hours (h); (3) RvD1 group: cells were treated with RvD1 (100 ng/mL) for 6 h; (4) RvD1 + LPS group: cells were pretreated with RvD1 (100 ng/mL) for 30 min and then treated with LPS (400 ng/mL) for 6 h. In some experiments, cells were pretreated with PD98059 (20 *μ*M) or PDTC (20 *μ*M) for 30 minutes, before being treated with RvD1 and LPS as described.

### 2.2. Permeability Assay

HUVECs (1 × 10^5^) were seeded on transwell filters (0.4-um pore size, Costar) in 24-well dishes and grown until they reached confluence. After treatment, the medium was replaced with serum-free medium. Fluorescein isothiocyanate (FITC)-dextran (Mr 40 000; Sigma) was then added to the upper chamber at a final concentration of 1 mg/mL. After 1 hour of incubation at 37°C, 100 *μ*L samples were taken from the lower chamber for fluorescence measurements. The fluorescent content of the samples were measured by using a spectrofluorimeter (*λ*EX 480 nm, *λ*EM 520 nm; Bio-Tek Synergy 2).

### 2.3. Immunofluorescence

After treatment, cells grown on coverslips were washed with PBS and fixed with 4% paraformaldehyde. Then, cells were washed twice with 1% PBS and permeabilized with 0.1% Triton X-100 for 5 min. The cells were blocked with 1% BSA in PBS for 30 min. For the staining of F-actin, cells were incubated with FITC-phalloidin (1 : 50; Enzo Life Sciences) for 60 min at room temperature. For zo-1 and occludin staining, cells were fixed with 4% paraformaldehyde and permeabilized with 0.05% Triton X-100 for 3 min. The cells were blocked with 1% BSA in PBS for 30 min and then incubated overnight at 4°C with the zo-1 antibody (1 : 50; Zymed, San Francisco, CA) or the occludin antibody (1 : 60; Zymed, San Francisco, CA) in a solution of 0.05% Tween-20 in TBS-goat serum (1 : 1). On the next day, the cell were washed with 0.05% Tween-20 in TBS and incubated with Dylight 594 AffiniPure Goat Anti-Rabbit IgG (1 : 400; EarthOx LLC San Francisco, CA) for 30 min at RT. The slides were incubated with antifade medium with DAPI for nuclear staining, and cell images were taken with the Olympus IX71 microscope.

### 2.4. Western Blotting

Proteins were extracted from scraped cells with RIPA buffer containing protease and phosphatase inhibitor cocktail tablets. Protein concentrations were assessed using the BCA protein assay kit. Samples were boiled in a 99°C heat block for 10 min and stored at −20°C until being used for immunoblotting. The samples (40 *μ*g) were separated by 8% SDS/PAGE, and the separated proteins were electrically transferred to PVDF membranes. The membranes were blocked for 1 hour with 5% nonfat dry milk in TBST (0.1% Tween-20 in TBS) at room temperature and then incubated overnight at 4°C with the following antibodies: polyclonal rabbit anti-zo-1 (1 : 400, Zymed, San Francisco, CA), occludin (1 : 500; Zymed, San Francisco, CA), ERK1/2 (1 : 1000; Cell Signal Technology), P-ERK1/2 (1 : 1000; Cell Signal Technology), I*κ*B*α* (1 : 1000; Cell Signal Technology), or rabbit anti-GAPDH (1 : 1000; Proteintech Group, Inc). The membranes were washed 3 times with TBS-T and incubated with goat anti-rabbit IgG (1 : 5000; Proteintech Group, Inc.) for 1 hour at RT. Protein bands were revealed by fluorography using ECL (enhanced chemiluminescence) reagents and quantified by the Image Lab image acquisition and analysis software (Bio-Rad).

### 2.5. Statistical Analyses

All data were expressed as the means ± s.e.m. and were analyzed with one-way analysis of variance followed by Newman-Keuls Multiple Comparison Test (GraphPad Prism (version 5 for Windows, San Diego, CA) software). Statistical significance was defined at *P* < 0.05.

## 3. Results

### 3.1. RvD1 Counteracted the LPS-Induced Increase in Endothelial Cell Permeability

**Figure 1:**
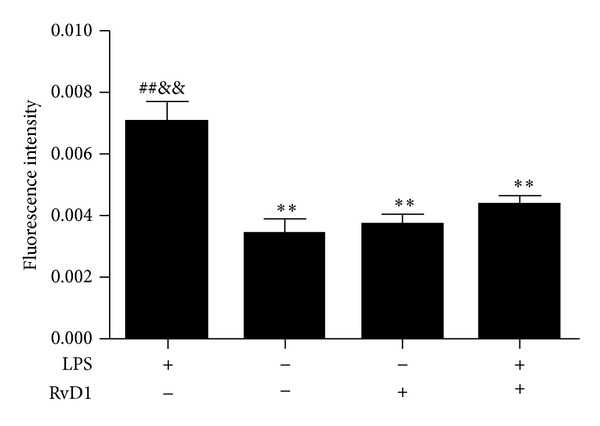
Effects of LPS and RvD1 on endothelial permeability measured by fluorescence intensity in HUVECs. Permeability was measured by determining the flux of FITC-dextran from the upper to the lower chamber. Data were expressed as mean ± s.e.m. (*n* = 3 per group). **P < 0.01 versus LPS group; ^##^ *P* < 0.01 versus control group; ^&&^ *P* < 0.01 versus RvD1 + LPS group.

The effects of LPS and RvD1 on endothelial TJ permeability in HUVECs were examined, as shown in Figure 1. LPS disrupted the permeability barrier in HUVECs (*P* < 0.01 Control versus LPS group) and the result is consistent with previous study [16]. RvD1 reduced the LPS-induced increase in permeability to a level comparable to that in the control group in HUVECs (*P* < 0.01 LPS group versus RvD1 + LPS group; Figure 1).

### 3.2. RvD1 Reversed the LPS-Induced Reorganization of the Actin Cytoskeleton and Tight Junctions and Increases zo-1 and Occludin Expression in HUVECs

**Figure 2:**
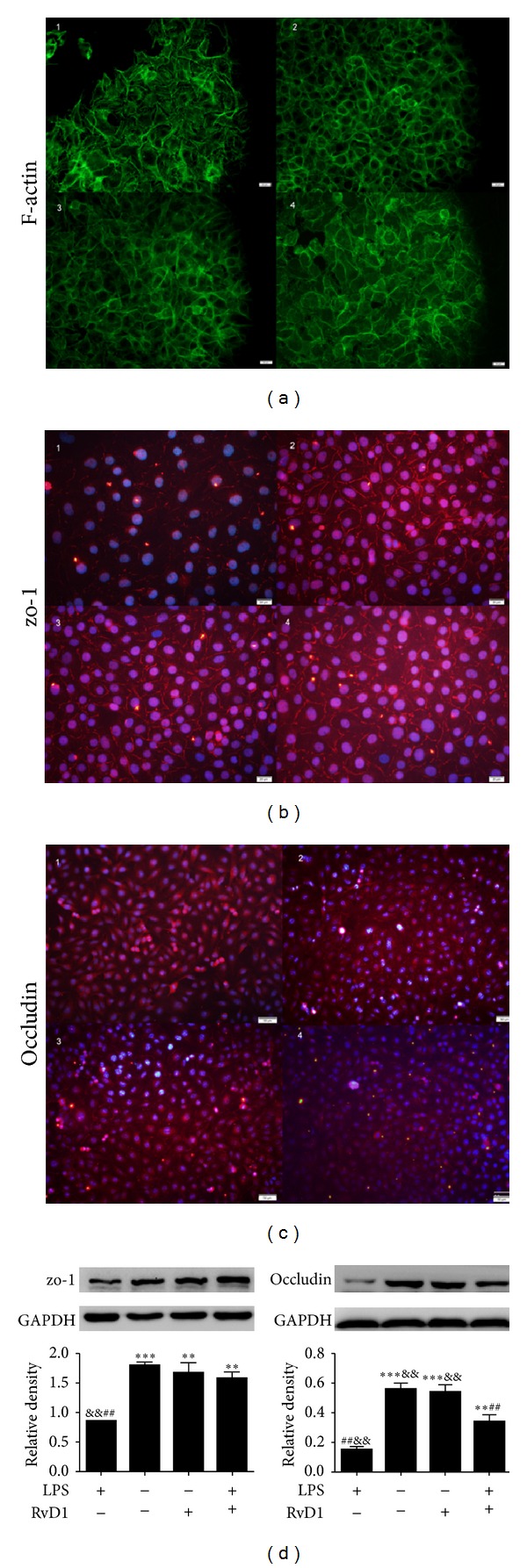
Effects of RvD1 and LPS on the junctional localization of zo-1, occludin, and F-actin as well as the expression of zo-1 and occludin in HUVECs. The locations of F-actin (a), zo-1 (b), and occludin (c) were detected by immunofluorescence. The protein expressions of zo-1 and occludin (d) were detected by western blotting. Data were expressed as mean ± s.e.m. (*n* = 3 per group). **P < 0.01, ***P < 0.001 versus LPS group; ^##^ *P* < 0.01 versus control group; ^&&^ *P* < 0.01 versus RvD1 + LPS group.

LPS has been shown to induce the redistribution of occludin and zo-1 from intercellular junctions [17, 18]. To study the effect of RvD1 on the reorganization and expression of zo-1, occludin and F-actin in LPS-induced endothelial cells, we treated HUVECs with RvD1 prior to LPS induction. As demonstrated in Figure 2, LPS induced a vast assembly of stress fibers and fragmentation of the occludin and zo-1 signals. Gaps were detected between cells, and the expression of zo-1 and occludin decreased significantly (*P* < 0.01 control versus LPS group; Figure 2(d)). Interestingly, RvD1 counteracted the LPS-induced formation of stress fibers (Figure 2(a)). The linear structure of the zo-1 and occludin signals appeared at the cell margins upon pretreatment with RvD1 (Figures 2(b) and 2(c)). In addition the expressions of zo-1 and occludin were increased (*P* < 0.01 LPS group versus RvD1 + LPS group; Figure 2(d)). The formation of stress fibers and the changes in tight junctions induced by LPS correlated with a decrease in endothelial barrier function, as measured by passage of FITC-dextran through HUVEC monolayers grown on Transwell filters.

### 3.3. Effects of RvD1 on ERK1/2 and I*κ*B*α* Signaling Pathways in LPS-Treated Endothelial Cells

**Figure 3:**
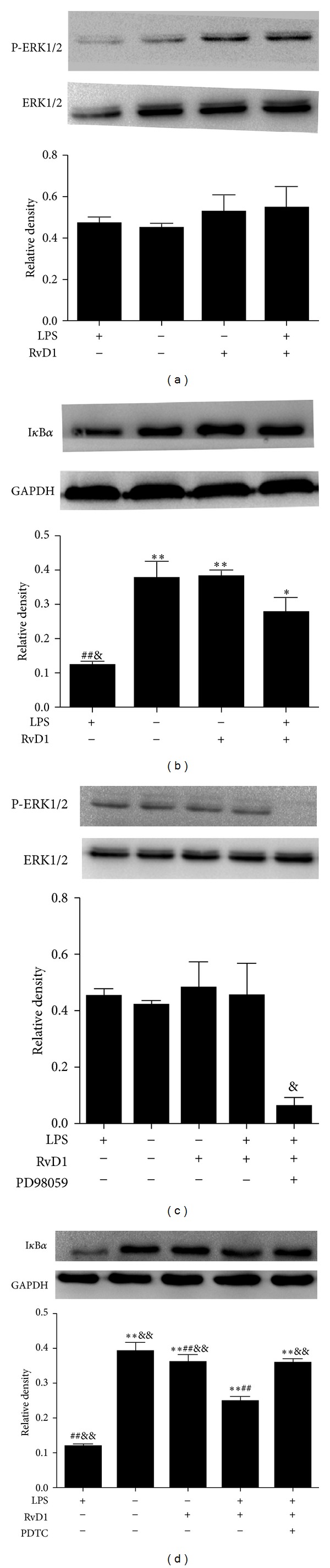
Effects of RvD1 and LPS on the ERK1/2 and I*κ*B*α* signaling pathways in HUVECs. (a) Effect of RvD1 and LPS on ERK1/2 phosphorylation. (b) Effect of RvD1 and LPS on I*κ*B*α* protein expression. (c) Effect of ERK inhibitor PD98059 on ERK1/2 phosphorylation in RvD1 + LPS group. (d) Effect of NF-*κ*B inhibitor PDTC on I*κ*B*α* protein expression in RvD1 + LPS group. Densitometric analysis of the protein levels of zo-1 and occludin were shown (*n* = 3). Data were expressed as mean ± s.e.m. **P < 0.01, *P < 0.05 versus LPS; ^##^ *P* < 0.01 versus control; ^&&^ *P* < 0.01, ^&^ *P* < 0.05 versus LPS + RvD1.

Our findings demonstrated that pretreatment with RvD1 increased I*κ*B*α* expression in LPS-induced HUVECs (Figure 3). There was no significant difference in ERK1/2 phosphorylation among the different groups of HUVECs (Figure 3(a)). Compared with the control group, the expression of I*κ*B*α* was reduced in the LPS-stimulated group (*P* < 0.01; Figure 3(b)). Compared with the LPS-treated group, the expression of I*κ*B*α* was significantly higher in RvD1 + LPS group (*P* < 0.05; Figure 3(b)). ERK inhibitor PD98059 significantly decreased ERK1/2 phosphorylation in RvD1 + LPS group (*P* < 0.05; Figure 3(c)). The NF-*κ*B inhibitor PDTC significantly increased I*κ*B*α* expression (*P* < 0.05; Figure 3(d)), which was consistent with the notion that PDTC can suppress the transfer of NF-*κ*B from the cytoplasm into the nucleus by inhibition of the I*κ*B*α* degradation.

### 3.4. RvD1 Prevented LPS-Induced Disruption of TJ by a Mechanism Dependent on I*κ*B*α* rather than ERK1/2

**Figure 4:**
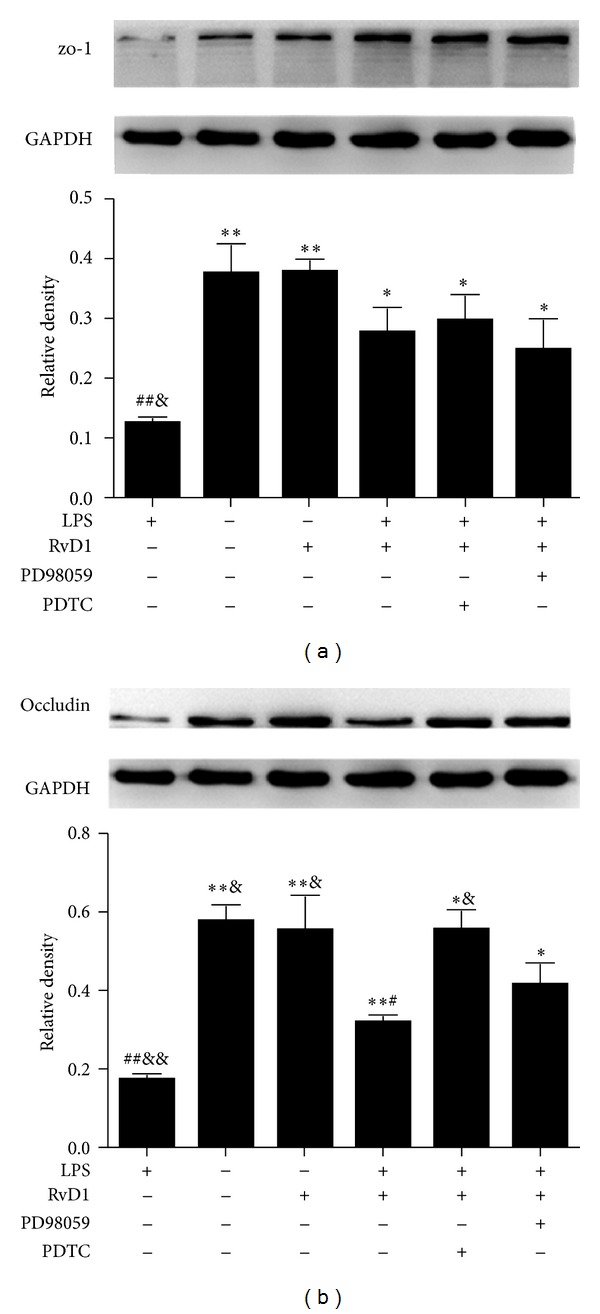
Effects of ERK1/2 and NF-*κ*B inhibitors on the expression of zo-1 and occludin in HUVECs. The protein expression of zo-1 (a) and occludin (b) were detected by western blotting. Densitometric analysis of the protein levels of zo-1 and occludin were shown (*n* = 3). Data was expressed as mean ± s.e.m. **P < 0.01, *P < 0.05 versus LPS group; ^##^ *P* < 0.01, ^#^ *P* < 0.05 versus control group; ^&&^ *P* < 0.01, ^&^ *P* < 0.05 versus RvD1 + LPS group.

As shown in Figure 4, RvD1 restored the expression of zo-1 and occludin in LPS-stimulated HUVECs. The NF-*κ*B inhibitor PDTC further enhanced the protective effects of RvD1 on restoration of occludin but not zo-1 expression in LPS-stimulated HUVECs (*P* < 0.05 RvD1 + LPS group versus RvD1 + LPS + PDTC group). However, ERK inhibitor PD98059 had no effect on the expression of TJ proteins in RvD1 + LPS group in HUVECs.

## 4. Discussion

Rapid changes in local blood vessel perfusion and permeability increase extravasation of circulating leucocytes and plasma proteins, which is the early phase of an acute inflammatory response [19]. Endothelial hyperpermeability plays a crucial role in vascular inflammation diseases, such as ischemia-reperfusion injury, thrombosis, cancer, and adult respiratory distress syndrome [20]. Increase in endothelial cell permeability can be caused by stimulation from a variety of inflammatory mediators including LPS, an endotoxin in the outer membrane of Gram-negative bacteria that stimulates mononuclear cells and neutrophils to produce immunoregulatory and proinflammatory cytokines [21]. Our results demonstrated that LPS induced an increase in endothelial cell permeability, which was in agreement with previous reports showing that LPS disrupts the permeability barrier of HUVECs [16]. RvD1, generated through sequential oxygenation of DHA, can reduce human polymorphonuclear leukocyte (PMN) transendothelial migration and inflammatory pain [22]. Interestingly, RvD1 reduces the levels of proinflammatory cytokines and increases the levels of ant-nflammatory cytokines [11]. RvD1 treatment leads to a significant reduction in the inflammatory cytokines IL-1*α*, IL-1*β*, and TNF-*α* [23]. We therefore examined whether RvD1 could protect cells from permeability barrier disruption induced by LPS. Our results showed that RvD1 indeed counteracted the LPS-induced increase in endothelial cell permeability. The reversal of LPS-induced barrier disruption by RvD1 seemed to be partly associated with the inhibition of inflammatory signaling pathway.

Inflammation leads to the loss of endothelial cell (EC) functional integrity and the formation of small gaps between ECs, which are a major cause of vascular leakage [24]. There is increasing evidence showing that zo-1 plays a crucial role in regulating TJ assembly. Studies have shown that TJ assembly is disrupted in cells with disrupted zo-1 expression [5–7]. Occludin is a 65-kDa protein located at the TJ and is the first transmembrane TJ protein identified [25]. Occludin also plays an important role in the paracellular barrier which mediates the flux of large macromolecules [26]. The main function of occludin involves TJ regulation [27, 28], although one study using occludin knockout mice and embryonic stem cells [29] has shown opposite results. Zo-1 and occludin are key molecules in paracellular permeability. Our results demonstrated that RvD1 protected the endothelial cells from barrier dysfunction, as measured by passage of FITC-dextran through HUVEC monolayers grown on Transwell filters after LPS stimulation. We also showed that RvD1 inhibited the redistribution of zo-1 and occludin and increases their expression in LPS-stimulated HUVECs. Exposure of HUEVCs to LPS significantly reduced zo-1 and occludin protein expressions, and this effect was reversed by pretreatment with RvD1. We next addressed the mechanism regarding how RvD1 influenced protein expression at TJ.

As a tight junction-associated cytoskeletal protein, zo-1 is involved in signal transduction and provides a link between occludin and the actin cytoskeleton [30]. The F-actin cytoskeleton determines cell shape and participates in the regulation of TJ proteins, which plays a major role in TJ barrier function and the regulation of paracellular pathways in different physiological and pathological states [31, 32]. It is thus not surprising that the disruption of this F-actin pool is associated with increased paracellular permeability. Recent research shows that incubation of human polymorphonuclears (PMNs) with RvD1 results in a decrease in actin polymerization [33]. Therefore, we examined whether RvD1 was able to reduce LPS-induced actin polymerization and reduce LPS-induced increase of permeability in HUVECs. Our findings revealed that LPS increased actin reorganization, which was consistent with the findings [34]. In addition, RvD1 indeed reduced LPS-induced actin polymerization. We thus concluded that RvD1 reversed the LPS-induced permeability of HUVEC by reducing actin polymerization.

Many inflammatory mediators are known to disrupt interendothelial junction assembly, thereby causing endothelial hyperpermeability. LPS, the interleukins IL-1, IL-3, and IL-4, tumor necrosis factor alpha (TNF-*α*), and interferon gamma (IFN-*γ*) are shown to influence TJ barrier function in epithelia and endothelia [35–37]. It is known that RvD1 significantly reduces the levels of IL-1*β* and IL-6 and increases the levels of IL-10 [11]. Moreover, the regulation of cytokines by RvD1 involves an increase in I*κ*B*α* expression, suggesting reduced NF-*κ*B activation in the lung [38]. It is well known that the activation of NF-*κ*B is regulated mainly by its subcellular localization, which is determined by the level of expression and the nucleocytoplasmic distribution of the I*κ*B proteins [39]. Although there are a number of I*κ*B proteins, I*κ*B*α* is the main regulator of NF-*κ*B activation, through a mechanism involving I*κ*B*α* degradation. LPS binds to Toll-like receptor 4 (TLR4) to activate NF-*κ*B and thus induces the transcription of proinflammatory mediators, leading to endothelial hyperpermeability [40, 41]. The activation of NF-*κ*B can inhibit the expression of TJ proteins [42]. Previous studies show that the absence of I*κ*B*α* increased NF-*κ*B activity after stimulation with LPS [43]. We therefore speculated that RvD1 might increase the expression of I*κ*B*α*, causing an increase in TJ protein expression. Our results showed that LPS reduced the expression of I*κ*B*α*, which was consistent with the report [44]. Importantly, when we pretreated cells with RvD1 before LPS stimulation, the expression of I*κ*B*α* was enhanced. The NF-*κ*B inhibitor PDTC further enhanced the protective effect of RvD1 on restoration of occludin but not zo-1 expression in LPS-stimulated HUVECs. From the above results, we concluded that RvD1 inhibited the LPS-induced redistribution of occludin and enhanced its expression in endothelial cells via a mechanism that partly involved the I*κ*B*α* pathway. However, RvD1 did not affect zo-1 through the I*κ*B*α* pathway, and the mechanism remains to be explored.

As an important regulator in signal transduction pathway, MAPK extracellular signal-regulated kinase (ERK) 1/2 (p44/p42, resp.) can activate many cell types during inflammation [45]. Treatment with LPS results in the loss of TJ integrity and an increase in paracellular permeability in corneal and alveolar epithelial monolayers [46, 47]. The activation of ERK1/2 is also found to be associated with TJ disruption through an H_2_O_2_-induced endothelial cell monolayer [13]. LPS treatment induces the disruption of epithelial barrier function in epithelial cells through a mechanism involving ERK1/2 phosphorylation [48] and induces the redistribution of occludin and zo-1 from intercellular junctions [18]. Interestingly, pretreatment of cells with RvD1 inhibits the activation of ERK1/2 and I*κ*B in the lung tissues of mice [12]. In view of these findings, we examined whether RvD1 could inhibit LPS-induced ERK1/2 activation and change endothelial permeability by protecting TJ integrity in endothelial cells and whether the changes in TJ protein expression could be reduced by inhibiting ERK1/2 activation. Unexpectedly, our studies showed that there were no differences in ERK1/2 phosphorylation in HUVECs between the experimental groups. In addition, the ERK1/2 inhibitor PD98059 had no effect on zo-1 and occludin protein expression in LPS-induced HUVECs. These results suggested that ERK1/2 signaling was not involved in the protective effect of RvD1 in LPS-induced redistribution of TJ proteins.

In summary, our findings revealed that RvD1 protected against endothelial barrier dysfunction by increasing the proteins expression of occuludin and zo-1 in LPS-induced HUVECs and RvD1 did this via a mechanism that involved the activation of I*κ*B*α* pathway but not ERK1/2 pathway. Notably, many inflammatory diseases involve changes in TJ protein expression and redistribution. All these were regarded as accelerating the course of inflammatory diseases, and research on tight junction proteins may thus become a new therapeutic strategy for inflammatory diseases.

## Abbreviations

ERK: Extracellular-signal regulated kinase
HUVEC: Human umbilical vein endothelial cell
IBD: Inflammatory bowel disease
IFN-*γ*: Interferon gamma
MDCK: Madin-Darby canine kidney
I*κ*B: Inhibitor of nuclear factor kappa B
MAPKs: Mitogen-activated protein kinases
RvD1: Resolvin D1
siRNA: Small interfering RNA
TJ: Tight junction
TNF-*α*: Tumor necrosis factor alpha.
